# Cold Weather Injuries Among the Active and Reserve Components of the U.S. Armed Forces, July 2019–June 2024

**Published:** 2024-11-20

**Authors:** Alexis L. Maule, Katherine S. Kotas, Kiara D. Scatliffe-Carrion, John F. Ambrose

**Affiliations:** 1 Disease Epidemiology Program, Defense Centers for Public Health–Aberdeen, Defense Health Agency, Aberdeen Proving Ground, MD

## Abstract

Over the 5-year surveillance period, from July 2019 through June 2024, the crude incidence rate of any cold weather injury was 31.1 per 100,000 person-years (p-yrs) for the active component and 6.4 per 100,000 persons for the reserve component. From July 2023 through June 2024, a total of 456 members of the active (n=403) and reserve (n=53) components of the U.S. Armed Forces had at least 1 cold weather injury. During the 2023-2024 cold season, the rates of any cold weather injury increased slightly for the active (31.1 per 100,000 p-yrs) and reserve (6.4 per 100,000 persons) components compared to the previous cold season. The rate of any cold weather injury varied among the services for the 2023-2024 cold season, with higher rates observed in the Army and the Marine Corps. Over the entire surveillance period, active component service member cold weather injury rates were higher among males, non-Hispanic Black or African Americans, and those under 20 years old.

**What are the new findings?:**

The crude incidence rate for cold weather injuries among all active component service members (31.1 per 100,000 p-yrs, 2023-2024), increased by 8.4% from the injury rate observed last season (28.7 per 100,000 p-yrs, 2022-2023) but remains unchanged from the rate observed during the 5-year surveillance period (31.1 per 100,000 p-yrs, 2019-2024). During the 2023-2024 cold season, active component Air Force personnel experienced their highest rate of any cold weather injury of the 5-year surveillance period.

**What is the impact on readiness and force health protection?:**

Despite the terminology, cold weather injuries can occur in a variety of conditions, and in much warmer temperatures than expected, particularly when operating or training in wet or aquatic environments. It is essential that both service members and their leadership understand the hazards in their environments, the health risks those hazards pose, and prevention strategies to combat them (e.g., weather-appropriate clothing, clean and dry socks and footwear, and proper protective gear for extremities).

## 1 BACKGROUND

Cold weather injuries are of significant military concern due to potential effects on service members (e.g., morbidity and potential disability) and the total force (e.g., adverse impacts on operations and costs of treatment).^1,2^ In response, the U.S. Armed Forces have developed, and are continually improving, their training, doctrine, procedures, and protective equipment and clothing to counter the threat of cold environments.^3,4,5,6^ Although these measures are effective when properly implemented, cold weather injuries continue to affect hundreds of service members each cold season due to exposures to both cold and wet environments.^7,8^

Cold weather injuries can be broadly categorized in 2 major groups: those with a central effect, and those primarily affecting the body’s periphery. Hypothermia occurs if the body cannot maintain a core temperature at or above 95°F. If skin temperatures reach 95°F, the body’s physiological response is initiated to minimize loss of heat and maintain the core temperature for vital organ protection.^9,10^ This response is achieved by decreasing blood flow to the extremities and redistributing warm blood to the core.^9,10,11^ Lack of blood flow to the extremities, even before a drop in core temperature, is the leading cause of peripheral cold injuries.

Initially, hypothermia may impair cognition (e.g., confusion, slurred speech, memory loss), heart rate, and breathing. Severe hypothermia can lead to loss of consciousness, pulmonary edema, coma, ventricular arrhythmias (including ventricular fibrillation), and asystole.^10,12,13^ Freezing atmospheric temperatures are not required to produce hypothermia, particularly when water immersion is involved. Because heat loss occurs 2 to 5 times faster in water compared to air, core body temperature can start to drop in water temperatures as warm as 80°F.^10^10

Peripheral cold injuries mainly affect the hands, feet, and face, and can be further classified as either freezing injuries, such as frostbite, or non-freezing injuries, such as immersion foot. Freezing peripheral injury is defined as the damage sustained by tissues when skin temperatures fall below freezing, most frequently affecting tissues of the ears, nose, cheeks, chin, fingers, and toes.^10,11,14,15,16^ A substantial proportion of patients with peripheral frostbite experience permanent changes in their microcirculation and disruption of localized nerve functions (e.g., reduced sensation in the affected area).^15^ Although most frostbite damage is minor, severe injury may lead to impaired functioning and inability to perform occupational tasks due to cold hypersensitivity, chronic ulceration, vasospasm, localized osteoarthritis, or chronic pain.^11,15,17^

Non-freezing peripheral injury includes a spectrum of localized injuries to the soft tissues, nerves, and vasculature of distal extremities that result from prolonged exposure to wet, cold (generally 32-59°F) conditions; the injury process is generally slower in warmer water.^10,11,14,18^ Although most non-freezing peripheral injuries involve feet, any body part can be affected by the condition, including hands.^19^ When immersion foot occurs, the foot becomes hyperemic (i.e., increased blood flow), painful, and swollen with continuous exposure; progression to blistering, decreased blood flow, ulceration, and gangrene is gradual.^11,18,20^

Environmental factors that increase risk of cold weather injury include prolonged outdoor exposure to temperatures 40°F and lower, wind speeds exceeding 5 miles per hour, high altitudes, geographic location, wet conditions due to rain or snow, and submersion in water.^19^ Situational factors that increase risk of cold weather injury include type of physical activity, inadequate shelter, and inappropriate clothing, including—specifically for non-freezing peripheral injuries of the foot—immobility, wet socks, and constrictive boots.^20,21,22^ Individual risk factors vary and include prior cold weather injury, dehydration, fatigue, improper acclimatization, inadequate nutrition, alcohol use, smoking, chronic disease (e.g., peripheral vascular disease, diabetes), and medications that impair compensatory responses (e.g., oral antihyperglycemics, beta-blockers, general anesthetic agents).^10,11,16,20,21,22^

Continuous surveillance of cold weather injuries is essential to understand the magnitude of the risk they pose, inform prevention efforts, and remind leaders of the hazards of training and operating in wet and cold environments. Department of Defense guidelines for reportable medical events (RMEs) require reporting of cases of hypothermia, freezing peripheral injuries (e.g., frostbite), and non-freezing peripheral injuries (e.g., immersion injuries, chilblains).^23^

Since 2004, *MSMR* has published annual updates on the incidence of cold weather injuries affecting U.S. Armed Force members for the 5 most recent cold seasons.^24^ The timing of these annual updates is intended to call attention to the recurring risks of such injuries as winter approaches in the Northern Hemisphere, where most members of the U.S. Armed Forces are assigned. This 2024 report addresses the occurrence of frostbite, immersion hand and foot injuries, and hypothermia during the cold seasons from July 2019 through June 2024.

## 2 METHODS

This surveillance population included all individuals who served in the active or reserve components of the U.S. Armed Forces at any time during the surveillance period of July 1, 2019 through June 30, 2024. For analysis purposes, a cold season was defined as July 1 through June 30 intervals so complete cold weather seasons could be represented in annual summaries and comparisons. Service members in the Space Force were classified separately from the Air Force for the 2022-2023 and 2023-2024 cold seasons as a result of complete data availability for the newly formed service; for previous cold seasons they were classified as Air Force.

Records of cold weather injuries for freezing peripheral injuries (i.e., frostbite), non-freezing peripheral injuries (i.e., immersion hand and foot injuries), and hypothermia were identified from 2 sources: 1) RMEs submitted to the Disease Reporting System internet (DRSi) and 2) diagnostic codes from inpatient and outpatient medical encounters in the Defense Medical Surveillance System and in-theater records from the Theater Medical Data Store. A cold weather injury case was defined by the presence of an RME or 1 of any qualifying International Classification of Diseases, 9th or 10th revision (ICD-9/ICD-10) codes in the first diagnostic position of a record of a health care encounter (**Table 1**).

To estimate the number of unique individuals who experienced a cold weather injury each cold season, and to avoid counting follow-up health care encounters, only 1 cold weather injury per individual per season was included in the counts of ‘any cold weather injury’. To count specific types of cold weather injury, namely frostbite, immersion hand and foot, and hypothermia cases, 1 of each type of cold weather injury per individual per season could be included in the counts of ‘all cold weather injuries’. For example, if an individual was diagnosed or reported with an immersion injury at 1 point during a cold season and then with frostbite later in the same cold season, each of those different injury types would be included in the injury-specific calculations. If a service member had multiple medical encounters for the same cold weather injury, only 1 encounter was used for analysis. The hospitalization encounter was prioritized over the ambulatory visit.

Annual seasonal incidence rates of cold weather injuries among active component service members (ACSMs) were calculated as incident cold weather injury diagnoses per 100,000 person-years (p-yrs) of service. Annual seasonal incidence rates of cold weather injuries among reservists were calculated as cases per 100,000 persons using the total number of reserve component service members for each cold season of the surveillance period. Counts of persons in the reserves were used as the denominator in these calculations because information on the start and end dates of active duty service periods of reserve component members is unavailable, so person-time cannot be accurately calculated.

Cold weather injuries are summarized by the locations where service members were treated for those injuries, identified by a Defense Medical Information System Identifier (DMIS ID) of a health care encounter. Because such injuries can occur during field training, temporary duty, or outside usual duty stations, DMIS ID was used as a proxy for the location where the cold weather injury occurred.

## 3 RESULTS

**2023-2024 cold season**

**Figure 1:**
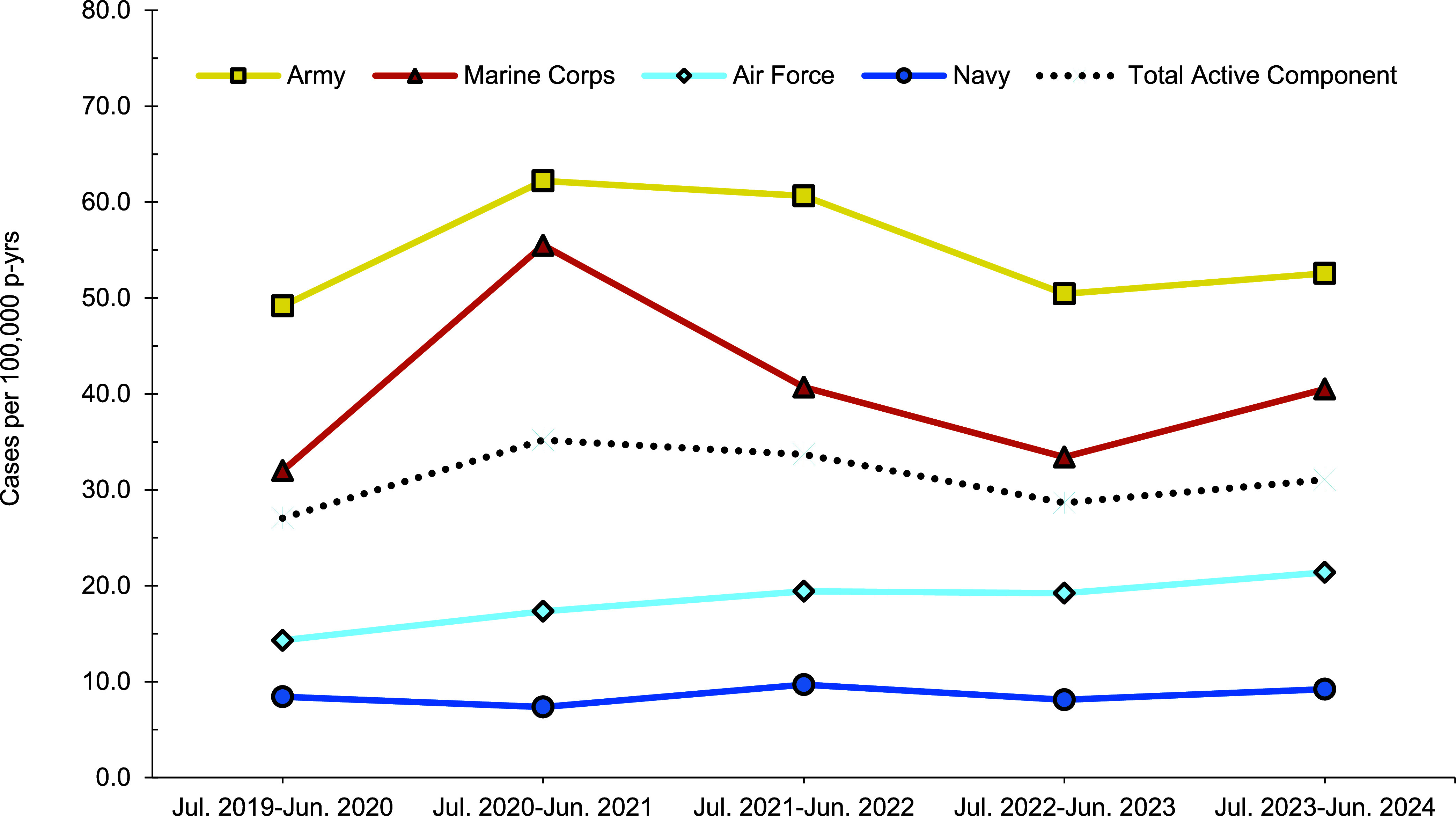
Annual Incidence Rates of Service Members Affected by Any Cold Injury (1 per person per year) by Service, Active Component, U.S. Armed Forces, July 2019–June 2024

**Figure 2:**
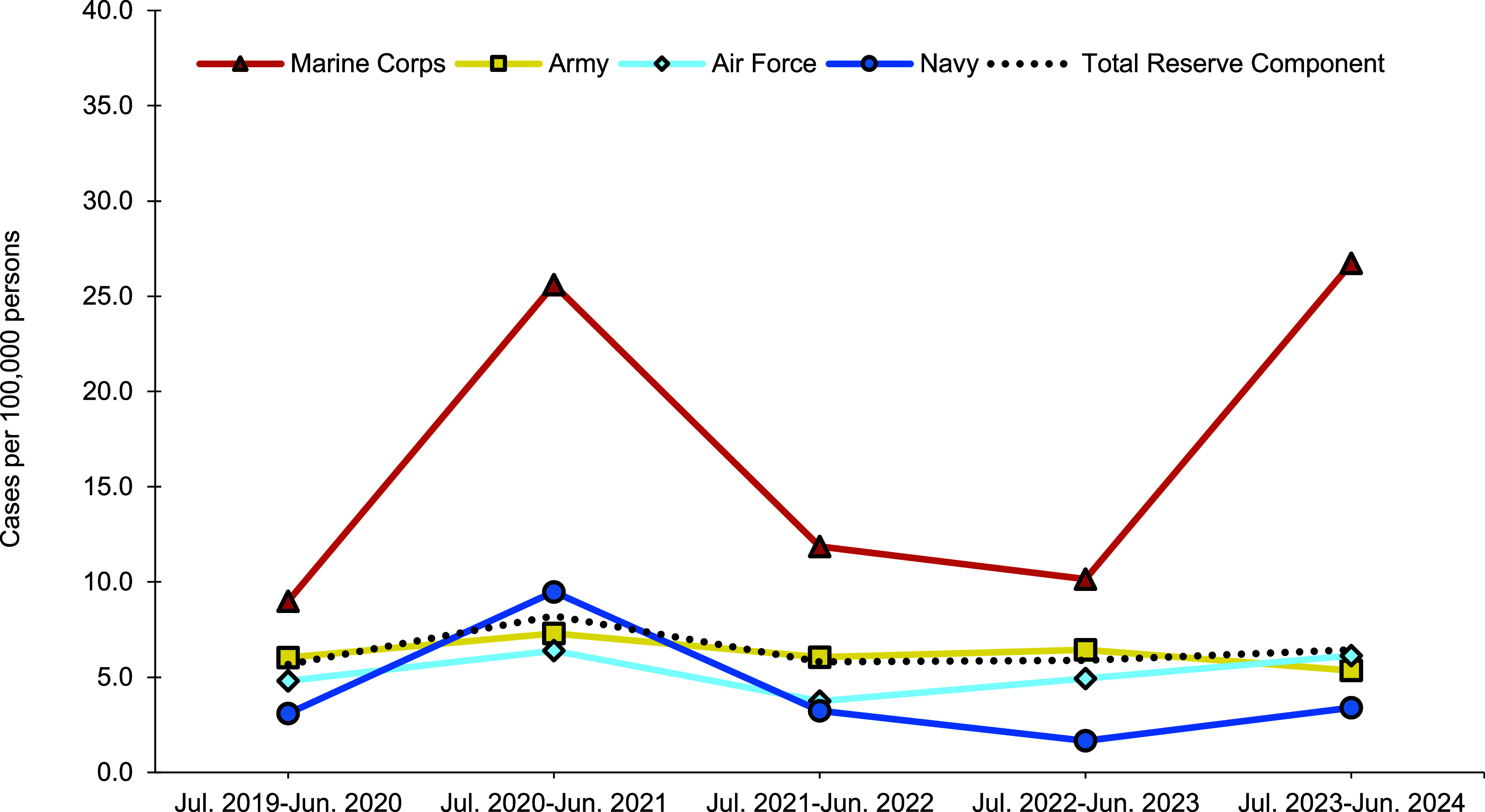
Annual Incidence Rates of Service Members Affected by Any Cold Injury (1 per person per year) by Service, Reserve Component, U.S. Armed Forces, July 2019–June 2024

From July 2023 through June 2024, a total of 456 members of the active (n=403) and reserve (n=53) components had at least 1 cold weather injury (**Table 2**, 1 per person per cold season). In the active component, soldiers had the highest rate of any cold weather injury (n=233, 52.6 per 100,000 p-yrs) during the 2023-2024 cold season, followed by members of the Marine Corps (n=68, 40.5 per 100,000 p-yrs), Air Force (n=67, 21.4 per 100,000 p-yrs), and Navy (n=30, 9.2 per 100,000 p-yrs). One active component Space Force member (11.1 per 100,000 p-yrs) and 4 active component Coast Guard members (10.2 per 100,000 p-yrs) were affected by cold weather injuries during the 2023-2024 cold season (**Table 2**, **Figure 1**). Within the reserve component, Army personnel accounted for over half of the cases (n=29, 5.3 per 100,000 persons) in 2023-2024 (**Table 2**, **Figure 2**), although Reservists in the Marine Corps (n=10, 26.7 per 100,000 persons) had higher rates of cold weather injuries.

Within the different services in 2023-2024, frostbite was the most common type of cold weather injury among active component Army (n=125, 45.5%), Navy (n=20, 66.7%), and Air Force (n=47, 67.1%) members (**Tables 3a, 3b, 3c**), whereas immersion injury was the most common type of cold weather injury among the Marine Corps active component (n=30, 44.1%) (**Table 3d**).

**Five cold seasons: July 2019–June 2024**

For all 5 cold seasons, the crude incidence rate of any cold weather injury for all ACSMs was 31.1 per 100,000 p-yrs (**Table 2**). For the most recent cold season, the crude incidence rate of any cold weather injury for all ACSMs increased by 8.4% (from 28.7 per 100,000 p-yrs in 2022-2023 to 31.1 per 100,000 p-yrs in 2023-2024) compared to the prior cold season (**Table 2**). Throughout the surveillance period, cold weather injury rates remained consistently higher among ACSMs in the Army and Marine Corps (**Figure 1**). During the 5-year surveillance period, the crude incidence rate of any cold weather injury for the reserve component was 6.4 per 100,000 persons (**Table 2**).

During the most recent current cold season, the crude incidence rates of any cold weather injury increased by 9.1% (from 5.9 per 100,000 persons in 2022-2023 to 6.4 per 100,000 persons in 2023-2024) compared to the prior cold season (**Table 2**).

Frostbite was the most common cold weather injury type among ACSMs during the first 3 cold seasons of the surveillance period, except among active component sailors, for whom hypothermia resulted in the highest cold weather injury rate during the 2020-2021 and 2021-2022 seasons (**Tables 3a, 3b, 3c, 3d, 3e**). The rate of immersion injury increased above the rate of frostbite injury for active component members of the Marine Corps during the 2022-2023 and 2023-2024 cold seasons (**Table 3d**). The rate of immersion injury also increased for Army active component members during the last 2 cold seasons, although the immersion injury rate in 2023-2024 (26.4 per 100,000 p-yrs) remained just below the rate of frostbite (28.2 per 100,000 p-yrs) (**Table 3a**).

During the 5-year surveillance period, overall rates of all cold weather injuries in the active component were generally higher among service members who were male, non-Hispanic Black or African American, and in the 2 youngest age groups (<20 and 20-24 years old) (**Tables 3a, 3b, 3c, 3d**). When specific types of cold weather injury were considered, male and non-Hispanic Black or African American personnel had higher rates of frostbite in comparison to other types of cold weather injury (**Tables 3a, 3b, 3c, 3d**). Among all cold weather injury cases reported within the active component during the 5-year period, the Marine Corps demonstrated the highest recruit cold weather injury rate (182.7 per 100,000 p-yrs). In all services, enlisted personnel had higher rates of cold weather injury compared to officers (**Tables 3a, 3b, 3c, 3d, 3e**).

Throughout the 5-year surveillance period, a total of 37 ACSMs (1.8% of the total) were hospitalized. Of the 37 active component hospitalizations, hypothermia (n=18) and frostbite (n=18) were equally represented, while only 1 hospitalization was due to immersion injury. The Army (n=25) and Marine Corps (n=7) accounted for a majority (86.5%) of hospitalized cases (data not shown).

**Cold weather injuries during deployments**

During the 5-year surveillance period a total of 65 cold weather injuries were diagnosed among service members deployed outside the U.S. (data not shown), of which 25 (38.5%) were frostbite, 30 (46.2%) were immersion injuries, and 10 (15.4%) were hypothermia. Approximately one-third (n=22) of all 65 deployment-associated cold weather injuries were diagnosed during the 2023-2024 cold season. Immersion injuries accounted for over three-quarters (n=18, 81.8%) of the cold weather injuries identified in service members deployed outside the U.S. during the 2023-2024 cold season.

**Figure 3:**
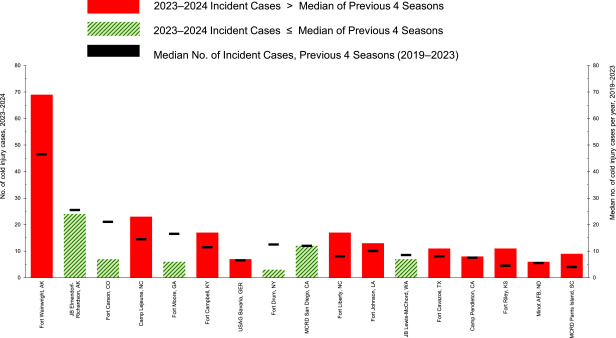
Annual Frequency (cold season 2023–2024) and Median Numbers (cold seasons 2019–2023) of Cold Injuries at Locations with at Least 25 Cold Injuries During the Surveillance Period, Active Component, U.S. Armed Forces, July 2019–June 2024

Cold weather injuries by geographic location During the 5-year surveillance period, 17 military locations reported at least 25 incidents of cold weather injury (1 per person per cold season) among ACSMs. **Figure 3** charts the 2023-2024 seasonal number of cold weather injuries (1 per person per year) in addition to the median case numbers for the previous 4 cold seasons for each of those 17 locations. The highest 5-year counts of incident cold weather injuries for seasons 2019-2024 were recorded at Fort Wainwright, AK (n=282), Joint Base Elmendorf-Richardson, AK (n=181), Fort Carson, CO (n=87), Marine Corps Base Camp Lejeune, NC (n=79), and Fort Moore, GA (n=75) (data not shown).

## 4 DISCUSSION

The overall rate of any cold weather injury in 2023-2024 for the active and reserve components increased by 8.4% and 9.1%, respectively, from the previous cold season. The rate increase for the current cold season was most pronounced in the Marine Corps active component (21.3%) and reserve component (163.3%). The Coast Guard and Space Force average less than 5 cases per year among their ACSMs, thus, small changes in the numbers of cases annually will result in abnormally large fluctuations in the injury rate. During the 2023-2024 cold season, the Air Force experienced its highest rate of any cold weather injury (21.4 per 100,000 p-yrs) over the 5-year surveillance period.

For the first 3 years of the 5-year surveillance period, the most common cold weather injury observed in ACSMs overall was frostbite, but in the last 2 years of the surveillance period immersion injury rates were higher for Marine Corps service members. This change in injury type prevalence could indicate a shift in environmental risk factors, but it does not signal a shift in injury severity. The long-term complications of non-freezing injury are similar to, and equally debilitating as, those produced by frostbite: hypersensitivity to cold, chronic pain, and severe pain induced by walking.^17,18,20^

Similar to previous *MSMR* reports, the highest cold weather injury rates were observed among male, younger age group, and non-Hispanic Black or African American service members.^8,24^ Higher rates of cold weather injury have also been noted among service members in the United Kingdom (U.K.) military with similar demographic characteristics.^21,25,26^ Differences in physiological responses to cold stress have been observed between different racial and ethnic groups, with individuals of African descent demonstrating greater vasoconstriction responses compared to individuals of Asian or Caucasian descent.^10,15,27^ Furthermore, signs and symptoms of cold weather injuries (e.g., skin redness, blotchy skin, waxy and white skin) may initially be more difficult to see on service members with skin of color.^28,29^ Service members, leadership, and medical personnel should be educated on the early signs and symptoms of cold weather injuries for a wide range of skin types.

When examining the service-specific demographic groups with increased rates, it should be noted that there were differences in the most frequently observed cold weather injury type. Younger marines had higher rates of hypothermia and younger soldiers had higher rates of frostbite compared to other cold weather injury types. These differences could indicate different situational risk factors for cold weather injury within the services, for example, training activities, occupational tasks, and geographic region. A study of U.K. service personnel noted that the most common situational risk factors for non-freezing peripheral injury were standing guard, as well as wet socks and boots.^21^ Unit leaders must be able to assess environmental, situational, and individual risk factors of their training and operational settings and understand how those factors increase risk of cold weather injuries for service members in their charge.

It should be noted that this analysis of cold weather injuries was unable to distinguish between injuries sustained during official military duties (e.g., training or operations) and those associated with unrelated or personal activities. In addition, the personnel files from the Defense Manpower Data Center used to calculate the population estimates for the active and reserve components as well as the demographic data presented in **Tables 3a, 3b, 3c, 3d, 3e** for the active component were unavailable for May and June 2024; the duty statuses of all service members, active and reserve, in April 2024 were assumed to be their May and June 2024 statuses. It is likely that some individuals in the U.S. Armed Forces both joined and left service during those months, and those movements are unaccounted for in the population estimates. Likewise, it is possible some time-varying demographics (e.g., age and rank) changed for individuals in May and June 2024, compared to April, and those shifts in categories are unaccounted. In all instances, however, the effect on the rates shown throughout the report should be minimal due to the large population size.

Cold weather injuries can be prevented by ensuring proper clothing, including layers that can be added or removed according to environmental conditions and physical activity, along with footwear that is non-constrictive, dry, and regularly changed if wet.^9,10,22^ Maintenance of proper hydration and nutrition, avoidance of long periods of sedentary or immobile positions, and planning for appropriate shelter and opportunities for re-warming are also important. Military training or mission requirements in cold and wet weather conditions can preclude immediate warm or dry shelter, ability to change wet or damp clothing, or even healthy physical activity.^2,3,11^ To prepare for all circumstances posing a threat for cold weather injury, service members should be cognizant of, and able to identify, signs of cold weather injury in addition to environmental, individual and situational risk factors. Service members should also be aware of protective measures for themselves and their fellow service members, whether during training, operations, combat, or recreational activities in wet or freezing conditions.

## Figures and Tables

**Table 1:** ICD-9 and ICD-10 Diagnostic Codes for Cold Weather Injuries

Case Classification	ICD-9	ICD-10^a^
Frostbite	991.0, 991.1, 991.2, 991.3	T33.*, T34.*
Immersion hand and foot	991.4	T69.0*
Hypothermia	991.6	T68.*

Abbreviation: ICD, International Classification of Diseases, 9th and 10th revisions.

^a^An asterisk (*) indicates that any subsequent digit / character is included.

**Table 2:** Annual Incidence of Service Members Affected by Any Cold Injury (1 per person per season), by Service and Component, July 2019–June 2024

Year	Army		Navy		Air Force		Marine Corps		Coast Guard		Space Force		All Services	
	No.	Rate^a^	No.	Rate^a^	No.	Rate^a^	No.	Rate^a^	No.	Rate^a^	No.	Rate^a^	No.	Rate^a^
Active component														
All years (2019-2024)	1,278	55.1	143	8.6	295	18.3	356	40.5	12	6.0	2	14.4	2,086	31.1
Jul. 2019–Jun. 2020	233	49.2	28	8.4	47	14.3	59	32.0	1	2.5	0	0.0	368	27.1
Jul. 2020–Jun. 2021	297	62.2	25	7.4	57	17.3	100	55.5	2	5.0	0	0.0	481	35.2
Jul. 2021–Jun. 2022	286	60.6	33	9.7	63	19.4	72	40.7	2	5.0	0	0.0	456	33.7
Jul. 2022–Jun. 2023	229	50.4	27	8.1	61	19.2	57	33.4	3	7.7	1	20.6	378	28.7
Jul. 2023–Jun. 2024	233	52.6	30	9.2	67	21.4	68	40.5	4	10.2	1	11.1	403	31.1
Reserve component														
All years (2019-2024)	175	6.2	13	4.2	48	5.2	34	16.5	4	12.2	0	0.0	274	6.4
Jul. 2019–Jun. 2020	35	6.0	2	3.1	9	4.8	4	9.0	0	0.0	0	0.0	50	5.7
Jul. 2020–Jun. 2021	42	7.3	6	9.5	12	6.4	11	25.6	1	15.1	0	0.0	72	8.2
Jul. 2021–Jun. 2022	34	6.0	2	3.2	7	3.7	5	11.9	2	30.3	0	0.0	50	5.8
Jul. 2022–Jun. 2023	35	6.4	1	1.7	9	4.9	4	10.2	0	0.04	0	0.0	49	5.9
Jul. 2023–Jun. 2024	29	5.3	2	3.4	11	6.1	10	26.7	1	15.4	0	0.0	53	6.4
Overall, active and reserve														
All years (2019-2024)	1,453		156		343		390		16		2		2,360	
Jul. 2019–Jun. 2020	268		30		56		63		1		0		418	
Jul. 2020–Jun. 2021	339		31		69		111		3		0		553	
Jul. 2021–Jun. 2022	320		35		70		77		4		0		506	
Jul. 2022–Jun. 2023	264		28		70		61		3		1		427	
Jul. 2023–Jun. 2024	262		32		78		78		5		1		456	

Abbreviations: No., number; Jul., July; Jun., June.

^a^For active component, rate is per 100,000 person-years. For reserve component, rate is per 100,000 persons.

**Table 3a:** Annual Incidence of Frostbite, Immersion Injury, and Hypothermia Among All Cold Injuries (1 type per person per year), Active Component, U.S. Army, July 2019–June 2024

	Frostbite		Immersion Injury		Hypothermia		All Cold Injuries	
	No.	Rate^a^	No.	Rate^a^	No.	Rate^a^	No.	Rate^a^
Total	734	31.6	434	18.7	186	8.0	1,354	58.4
Sex								
Male	641	32.7	390	19.9	162	8.3	1,193	60.9
Female	93	25.8	44	12.2	24	6.7	161	44.7
Race and ethnicity								
White, non-Hispanic	247	20.2	199	16.3	87	7.1	533	43.6
Black, non-Hispanic	343	73.2	161	34.4	57	12.2	561	119.8
Hispanic	89	21.8	58	14.2	24	5.9	171	41.9
Other	55	24.9	16	7.3	18	8.2	89	40.3
Age, y								
<20	68	46.7	52	35.7	18	12.4	138	94.8
20–24	322	45.6	227	32.1	97	13.7	646	91.4
25–29	154	27.7	82	14.7	44	7.9	280	50.4
30–34	95	25.3	44	11.7	13	3.5	152	40.5
35–39	54	19.3	13	4.6	9	3.2	76	27.1
40–44	23	14.9	9	5.8	3	1.9	35	22.6
45+	18	17.7	7	6.9	2	2.0	27	26.5
Rank								
Recruit trainee	8	17.5	7	15.3	5	11.0	20	43.8
Enlisted	647	35.8	388	21.5	165	9.1	1,200	66.4
Officer	79	17.0	39	8.4	16	3.4	134	28.8
Occupation								
Infantry/artillery/armor/combat engineering	302	52.1	216	37.3	96	16.6	614	106.0
Motor transport	26	36.6	12	16.9	4	5.6	42	59.2
Repair/engineering	110	23.9	63	13.7	26	5.7	199	43.3
Communications/intelligence	151	26.2	83	14.4	28	4.9	262	45.5
Health care	47	20.7	17	7.5	13	5.7	77	33.9
Other	98	24.1	43	10.6	19	4.7	160	39.3
Cold season (July–June)								
2019–2020	115	24.3	94	19.8	46	9.7	255	53.8
2020–2021	180	37.7	81	17.0	38	8.0	299	62.6
2021–2022	190	40.3	67	14.2	38	8.1	295	62.5
2022–2023	124	27.3	75	16.5	31	6.8	230	50.7
2023–2024	125	28.2	117	26.4	33	7.4	275	62.1

Abbreviations: No., number; y, years.

^a^Rate per 100,000 person-years.

**Table 3b:** Annual Incidence of Frostbite, Immersion Injury, and Hypothermia Among All Cold Injuries (1 type per person per year), Active Component, U.S. Navy, July 2019–June 2024

	Frostbite		Immersion Injury		Hypothermia		All Cold Injuries	
	No.	Rate^a^	No.	Rate^a^	No.	Rate^a^	No.	Rate^a^
Total	63	3.8	30	1.8	50	3.0	143	8.6
Sex								
Male	56	4.2	27	2.0	44	3.3	127	9.6
Female	7	2.0	3	0.9	6	1.8	16	4.7
Race and ethnicity								
White, non-Hispanic	32	3.9	12	1.5	28	3.4	72	8.7
Black, non-Hispanic	13	4.9	9	3.4	8	3.0	30	11.3
Hispanic	10	3.5	4	1.4	7	2.4	21	7.3
Other	8	2.7	5	1.7	7	2.4	20	6.8
Age, y								
<20	6	6.2	6	6.2	7	7.2	19	19.7
20–24	19	3.8	11	2.2	23	4.7	53	10.7
25–29	17	4.2	3	0.7	15	3.7	35	8.7
30–34	5	1.7	3	1.0	3	1.0	11	3.8
35–39	7	3.3	2	0.9	2	0.9	11	5.2
40–44	4	3.6	4	3.6	0	0.0	8	7.2
45+	5	7.6	1	1.5	0	0.0	6	9.1
Rank								
Recruit trainee	1	4.1	1	4.1	1	4.1	3	12.4
Enlisted	47	3.4	26	1.9	45	3.3	118	8.6
Officer	15	5.4	3	1.1	4	1.4	22	7.9
Occupation								
Infantry/artillery/armor/combat engineering	3	2.9	0	0.0	3	2.9	6	5.8
Motor transport	1	1.5	0	0.0	17	25.6	18	27.1
Repair/engineering	17	2.3	9	1.2	12	1.6	38	5.2
Communications/intelligence	8	3.0	8	3.0	3	1.1	19	7.1
Health care	18	10.3	0	0.0	3	1.7	21	12.1
Other	16	4.9	13	4.0	12	3.7	41	12.5
Cold season (July–June)								
2019–2020	14	4.2	6	1.8	8	2.4	28	8.4
2020–2021	9	2.6	3	0.9	13	3.8	25	7.4
2021–2022	12	3.5	8	2.3	13	3.8	33	9.7
2022–2023	8	2.4	7	2.1	12	3.6	27	8.1
2023–2024	20	6.2	6	1.8	4	1.2	30	9.2

Abbreviations: No., number; y, years.

^a^Rate per 100,000 person-years.

**Table 3c:** Annual Incidence of Frostbite, Immersion Injury, and Hypothermia Among All Cold Injuries (1 type per person per year), Active Component, U.S. Air Force, July 2019–June 2024

	Frostbite		Immersion Injury		Hypothermia		All Cold Injuries	
	No.	Rate^a^	No.	Rate^a^	No.	Rate^a^	No.	Rate^a^
Total	233	14.4	25	1.5	43	2.7	301	18.7
Sex								
Male	206	16.2	22	1.7	35	2.8	263	20.7
Female	27	7.9	3	0.9	8	2.3	38	11.1
Race and ethnicity								
White, non-Hispanic	117	12.5	12	1.3	22	2.3	151	16.1
Black, non-Hispanic	58	26.0	4	1.8	6	2.7	68	30.5
Hispanic	34	12.9	5	1.9	10	3.8	49	18.6
Other	24	12.6	4	2.1	5	2.6	33	17.4
Age, y								
<20	19	26.8	4	5.6	3	4.2	26	36.6
20–24	114	25.0	12	2.6	17	3.7	143	31.4
25–29	45	11.2	4	1.0	11	2.7	60	15.0
30–34	25	8.3	0	0.0	8	2.6	33	10.9
35–39	19	8.1	5	2.1	1	0.4	25	10.6
40–44	4	3.9	0	0.0	3	2.9	7	6.7
45+	7	15.7	0	0.0	0	0.0	7	15.7
Rank								
Recruit trainee	0	0.0	0	0.0	1	5.0	1	5.0
Enlisted	213	16.6	21	1.6	35	2.7	269	21.0
Officer	20	6.4	4	1.3	7	2.2	31	10.0
Occupation								
Infantry/artillery/armor/combat engineering	9	71.1	0	0.0	0	0.0	9	71.1
Motor transport	0	0.0	0	0.0	0	0.0	0	0.0
Repair/engineering	94	19.1	8	1.6	8	1.6	110	22.3
Communications/intelligence	36	10.5	1	0.3	6	1.7	43	12.5
Health care	9	6.1	1	0.7	2	1.4	12	8.1
Other	85	14.1	15	2.5	27	4.5	127	21.0
Cold season (July–June)								
2019–2020	38	11.6	2	0.6	8	2.4	48	14.6
2020–2021	47	14.3	1	0.3	9	2.7	57	17.3
2021–2022	53	16.3	5	1.5	6	1.8	64	19.7
2022–2023	48	15.1	7	2.2	7	2.2	62	19.5
2023–2024	47	15.0	10	3.2	13	4.2	70	22.3

Abbreviations: No., number; y, years.

^a^Rate per 100,000 person-years.

**Table 3d:** Annual Incidence of Frostbite, Immersion Injury, and Hypothermia Among All Cold Injuries (1 type per person per year), Active Component, U.S. Marine Corps, July 2019–June 2024

	Frostbite		Immersion Injury		Hypothermia		All Cold Injuries	
	No.	Rate^a^	No.	Rate^a^	No.	Rate^a^	No.	Rate^a^
Total	156	17.7	119	13.5	84	9.5	359	40.8
Sex								
Male	149	18.7	109	13.6	71	8.9	329	41.2
Female	7	8.6	10	12.3	13	16.0	30	36.9
Race and ethnicity								
White, non-Hispanic	72	14.5	68	13.7	38	7.7	178	35.9
Black, non-Hispanic	45	51.2	9	10.2	23	26.2	77	87.7
Hispanic	29	13.1	31	14.0	14	6.3	74	33.3
Other	10	13.3	11	14.7	9	12.0	30	40.0
Age, y								
<20	24	20.4	61	51.8	22	18.7	107	90.8
20–24	90	21.5	46	11.0	46	11.0	182	43.4
25–29	26	16.5	9	5.7	9	5.7	44	27.9
30–34	13	16.1	2	2.5	4	4.9	19	23.5
35–39	3	4.8	1	1.6	1	1.6	5	8.0
40–44	0	0.0	0	0.0	2	7.2	2	7.2
45+	0	0.0	0	0.0	0	0.0	0	0.0
Rank								
Recruit trainee	2	6.3	42	132.3	14	44.1	58	182.7
Enlisted	134	18.1	68	9.2	64	8.6	266	35.9
Officer	20	18.5	9	8.3	6	5.5	35	32.3
Occupation								
Infantry/artillery/armor/combat engineering	92	49.1	15	8.0	21	11.2	128	68.3
Motor transport	2	4.6	2	4.6	1	2.3	5	11.6
Repair/engineering	12	5.7	8	3.8	8	3.8	28	13.2
Communications/intelligence	24	11.4	10	4.7	8	3.8	42	19.9
Health care	0	0.0	0	0.0	0	0.0	0	0.0
Other	26	11.5	84	37.1	46	20.3	156	68.8
Cold season (July–June)								
2019–2020	26	14.1	15	8.1	18	9.8	59	32.0
2020–2021	55	30.5	25	13.9	20	11.1	100	55.5
2021–2022	33	18.7	23	13.0	18	10.2	74	41.9
2022–2023	21	12.3	26	15.2	11	6.4	58	34.0
2023–2024	21	12.5	30	17.9	17	10.1	68	40.5

Abbreviations: No., number; y, years.

^a^Rate per 100,000 person-years.

**Table 3e:** Annual Incidence of Frostbite, Immersion Injury, and Hypothermia Among All Cold Injuries (1 type per person per year), Active Component, U.S. Coast Guard, July 2019–June 2024

	Frostbite		Immersion Injury		Hypothermia		All Cold Injuries	
	No.	Rate^a^	No.	Rate^a^	No.	Rate^a^	No.	Rate^a^
Total	7	3.5	0	0.0	5	2.5	12	6.0
Sex								
Male	7	4.2	0	0.0	4	2.4	11	6.5
Female	398,309	322,329	0	0.0	1	3.2	1	3.2
Race and ethnicity								
White, non-Hispanic	5	4.0	0	0.0	3	2.4	8	6.3
Black, non-Hispanic	0	0.0	0	0.0	2	19.6	2	19.6
Hispanic	1	3.3	0	0.0	0	0.0	1	3.3
Other	1	3.1	0	0.0	0	0.0	1	3.1
Age, y								
<20	0	0.0	0	0.0	0	0.0	0	0.0
20–24	0	0.0	0	0.0	2	4.8	2	4.8
25–29	2	5.0	0	0.0	2	5.0	4	9.9
30–34	1	2.6	0	0.0	0	0.0	1	2.6
35–39	1	2.6	0	0.0	1	2.6	2	5.3
40–44	3	13.2	0	0.0	0	0.0	3	13.2
45+	0	0.0	0	0.0	0	0.0	0	0.0
Rank								
Recruit trainee	0	0.0	0	0.0	0	0.0	0	0.0
Enlisted	6	3.9	0	0.0	5	3.3	11	7.2
Officer	1	2.3	0	0.0	0	0.0	1	2.3
Occupation								
Infantry/artillery/armor/combat engineering	0	0.0	0	0.0	0	0.0	0	0.0
Motor transport	1	3.5	0	0.0	2	7.0	3	10.5
Repair/engineering	2	3.2	0	0.0	1	1.6	3	4.8
Communications/intelligence	1	3.4	0	0.0	2	6.8	3	10.2
Health care	0	0.0	0	0.0	0	0.0	0	0.0
Other	3	4.0	0	0.0	0	0.0	3	4.0
Cold season (July–June)								
2019–2020	1	2.5	0	0.0	0	0.0	1	2.5
2020–2021	1	2.5	0	0.0	1	2.5	2	5.0
2021–2022	1	2.5	0	0.0	1	2.5	2	5.0
2022–2023	2	5.1	0	0.0	1	2.6	3	7.7
2023–2024	2	5.1	0	0.0	2	5.1	4	10.2

Abbreviations: No., number; y, years.

^a^Rate per 100,000 person-years.

## AUTHORS' AFFILIATION

Disease Epidemiology Program, Defense Centers for Public Health–Aberdeen, Defense Health Agency, Aberdeen Proving Ground, MD
